# Electrochemical Detection of Hydrazine Using Poly(dopamine)-Modified Electrodes

**DOI:** 10.3390/s16050647

**Published:** 2016-05-05

**Authors:** Ji Young Lee, Truc Ly Nguyen, Jun Hui Park, Byung-Kwon Kim

**Affiliations:** 1 Department of Chemistry, Korea Advanced Institute of Science and Technology, Daejeon 34141, Korea; berry1504@kaist.ac.kr; 2 Department of Chemistry, Sookmyung Women’s University, Seoul 04310, Korea; trucly162@sookmyung.ac.kr; 3 Department of Chemistry Education and Institute of Fusion Science, Chonbuk National University, Jeonju 54896, Korea

**Keywords:** hydrazine, detection, indium tin oxide electrode, dopamine, poly(dopamine) film, cyclic voltammetry

## Abstract

We have developed a simple and selective method for the electrochemical detection of hydrazine (HZ) using poly(dopamine) (pDA)-modified indium tin oxide (ITO) electrodes. Modification with pDA was easily achieved by submerging the ITO electrode in a DA solution for 30 min. The electrocatalytic oxidation of HZ on the pDA-modified ITO electrode was measured by cyclic voltammetry. In buffer solution, the concentration range for linear HZ detection was 100 µM–10 mM, and the detection limit was 1 µM. The proposed method was finally used to determine HZ in tap water to simulate the analysis of real samples. This method showed good recovery (94%–115%) and was not affected by the other species present in the tap water samples.

## 1. Introduction

Hydrazine (HZ, N_2_H_4_) is an inorganic compound that exists as a volatile and flammable colorless liquid. HZ and its derivatives are mainly used in fuel-cell technology [1] and as propellants in rocket fuel, pesticides for agriculture, intermediates in photoprinting and pharmaceuticals, and deoxidizers in boilers. HZ is considered hazardous and is classified as a carcinogen by the US Environmental Protection Agency (EPA) [2] because it can cause chromosome aberrations and negatively affect the lungs, liver, spleen, thyroid gland, and central nervous system. Therefore, methods for the detection and monitoring systems for HZ should be developed to meet the demands of both industries and environmental groups. To date, various analytical methods such as titrimetry [3], chromatography [4], spectrophotometry [5], flow injection analysis with chemiluminescence [6], potentiometry [7], and amperometry [8,9,10,11] have been used for HZ detection. Among these techniques, electrochemical methods have advantages of simplicity, high sensitivity, rapid response, and low cost. To detect HZ using electrochemical methods, electrodes should be modified using special materials with electrocatalytic activities for the electrochemical oxidation of HZ to overcome the limitations associated with the high overpotential and sluggish kinetics of HZ in electrochemical reactions [12]. To achieve this, many studies have suggested the modification of electrodes by employing nanoparticles (e.g., Au, Ti, Pd, ZnO, Co, Sn, ZrO_2_, Bi, and Ce) [13,14,15,16,17,18,19,20,21], CoPC nanoparticles [22], manganese hexacyanoferrate [9], CoOOH nanosheets [12], zinc oxide nanowires [23], 4-pyridyl hydroquinone [24], biomolecules [25], and branched hierarchical ZnO nanorod arrays [26]. However, in many cases, creating these materials and using them to modify the electrode requires several preparation steps, which not only increases the cost of implementing the technique but also decreases its reproducibility. Therefore, simple and reproducible modification methods are urgently needed. To meet this requirement, in this study we adopted poly(dopamine) (pDA) to modify electrodes to detect HZ. We used pDA as a modifier or an electrocatalyst [19,27,28,29,30,31], because pDA can be simply and quickly formed on many substrates in basic solutions [32,33,34,35].

Indium tin oxide (ITO) electrodes are mainly used in display and solar cell applications because of their good electrical conductivity, optical transparency, and low cost. These electrodes are particularly applicable to electrochemical sensors because of their low background current and high reproducibility [36,37]. Another characteristic property of ITO electrodes is that they have lower catalytic activity than other metal (e.g., Pt, Au, and Pd) electrodes, which can suppress interfering electrochemical reactions during measurements. Although the low catalytic activity of ITO electrodes can also decrease the target (e.g., HZ) current signal, this problem can be resolved by modifying the electrodes with specific materials.

**Figure 1:**
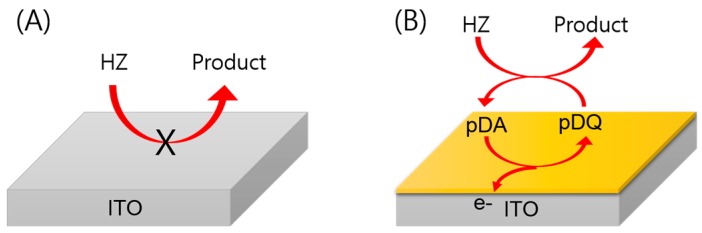
(**A**) Electrochemical oxidation of HZ rarely occurs on a bare ITO electrode; (**B**) HZ can be readily detected because of the electrochemical-chemical (EC) reaction on the pDA-ITO electrode.

Herein, we propose a pDA-modified ITO electrode for sensitive detection of HZ in solution. Because the formation of pDA films from DA solution is spontaneous and rapid, pDA-modified ITO electrodes were very easily obtained by submerging the electrodes in a DA solution for 30 min [27,33,38]. It is already known that DA polymerizes spontaneously to pDA in basic solution; in this process, DA is first oxidized to dopaminequinone, followed by intramolecular cyclization and rearrangement. Next, the product, leukodopaminechrome, is oxidized to dopaminechrome. Finally, dopaminechrome polymerizes to pDA on an ITO electrode. We used the resulting pDA-ITO electrode to detect HZ. As shown in Figure 1, the electrochemical reaction occurred on the pDA-ITO electrode when an appropriate potential was applied. In this process, pDA on the ITO electrode is first oxidized to poly(dopamine-o-quinone) (pDQ), generating an anodic current, and it is then reduced back to pDA by HZ, which is a strong reducing agent. Because of this redox cycling reaction, very high anodic currents can be obtained.

## 2. Materials and Methods

### 2.1. Chemicals and Instruments

Dopamine hydrochloride, HZ, KCl, Na_3_PO_4_, CaCO_3_, ZnCl_2_, MgSO_4_, MnCl_2_, FeCl_2_, CoCl_2_, and Tris were acquired from Sigma–Aldrich (Yongin, Korea). Unless otherwise indicated, all reagents were used as received. Tris buffer was prepared using 0.05 M Tris, 0.138 M NaCl, and 0.0027 M KCl. All aqueous solutions were made with ultrapure water (>18 MΩ·cm, Millipore, Darmstadt, Germany). ITO electrodes (30 Ω) were purchased from Samsung Corning (Daegu, Korea). Electrochemical measurements were performed using a CHI617B device (CH Instruments, Austin, TX, USA).

### 2.2. Electrochemical Measurement

A standard three-electrode cell with an ITO working electrode, a Au wire counter electrode, and a Ag/AgCl reference electrode (3 M KCl) was used. The area of the ITO working electrode was 0.28 cm^2^. The ITO working electrodes were sequentially washed in acetone, ethanol, and ultrapure water with ultrasonication for 15 min. The pDA-ITO working electrodes were obtained by submerging the cleaned ITO electrodes in Tris buffer containing 1 mM DA for 30 min. Subsequently, the pDA-ITO electrodes were washed with distilled water. All HZ-detection experiments were conducted in Tris buffer using pDA-ITO electrodes. All experiments were carried out at room temperature. Air-saturated solutions were used without any further preparation step (e.g., Ar bubbling).

## 3. Results and Discussion

### 3.1. Electrochemical Detection of HZ

**Figure 2:**
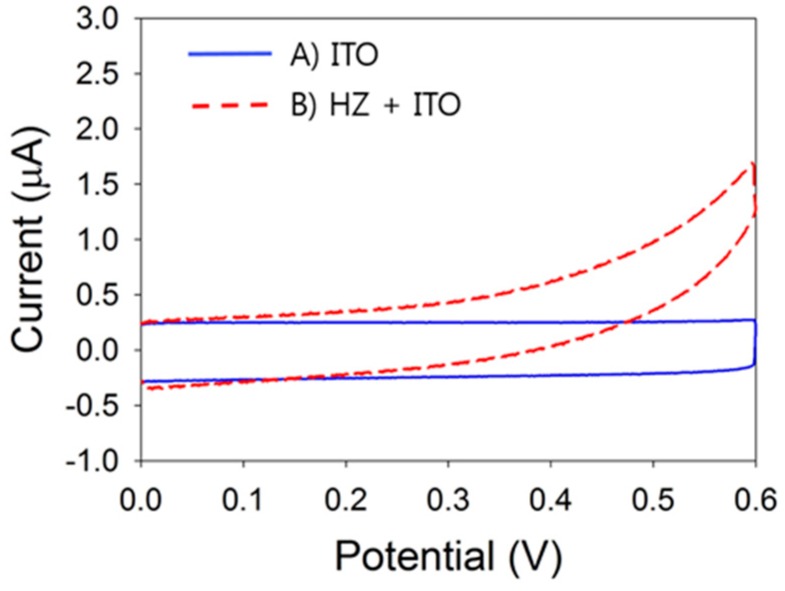
Cyclic voltammograms of (A) Tris buffer (pH 8) and (B) 1 mM·HZ in Tris buffer (pH 8) using an ITO electrode at a scan rate 50 mV/s.

The electrochemical oxidation properties of HZ were examined on a bare ITO electrode using commercially available Tris buffer (pH 8) (Figure 2). In the absence of HZ, only the capacitive current of the bare ITO electrode was measured (Figure 2A). In the presence of 1 mM·HZ, a sluggish oxidation peak was observed because of the slow electron transfer between HZ and the bare ITO electrode (Figure 2B). This slow electron transfer leads to a low anodic current and prevents sensitive detection. Thus, for HZ sensing applications, the HZ oxidation current must be increased.

**Figure 3:**
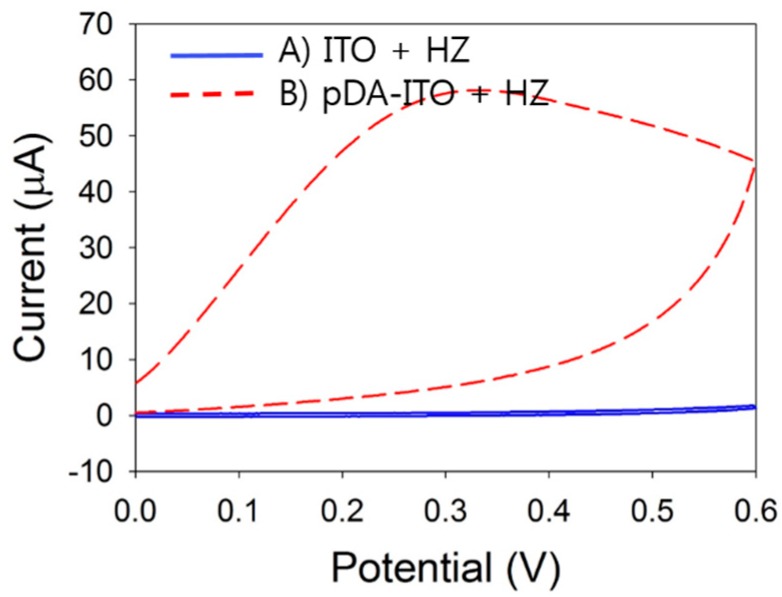
Cyclic voltammograms of 1 mM·HZ in Tris buffer with (A) an ITO electrode and (B) a pDA-ITO electrode at a scan rate 50 mV/s.

Spontaneous formation of a pDA film was achieved on a bare ITO electrode by simply immersing the electrode in Tris buffer containing 1 mM DA for 30 min. The resulting pDA film can be easily oxidized at the ITO electrode [34]. Previously, we suggested a DA-detection method using pDA films on ITO electrodes in the presence of HZ, wherein the oxidation current of pDA increased proportionally to the concentration of DA [35]. By inverting the concept underlying the previously described method, pDA films can be used as electrocatalytic mediators for sensitive detection of HZ oxidation. For this purpose, HZ oxidation was performed on pDA film-modified ITO electrodes in Tris buffer containing 1 mM·HZ. Unlike the case with the bare ITO electrode, we observed a large peak-shaped HZ oxidation current of approximately 60 μA at roughly 0.3 V in the modified electrode (Figure 3).

**Figure 4:**
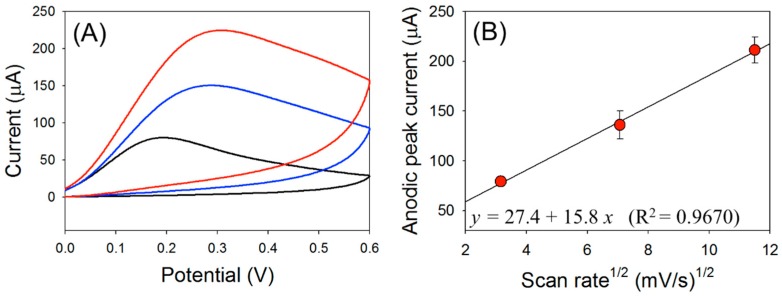
(**A**) Cyclic voltammograms of 10 mM·HZ in Tris buffer with pDA-ITO electrodes at a scan rate of 10 mV/s (black line), 50 mV/s (blue line), and 100 mV/s (red line); (**B**) Dependence of the anodic peak current on the square root of the scan rate.

This 60 μA current represents an increase of nearly 40-fold compared with that with the bare ITO electrode (*ca.* 0.15 μA). These results indicate that HZ oxidation by the pDA film could be applied for sensitive detection of HZ. Two reactions occurred simultaneously at the pDA-ITO electrode: the first is electrochemical oxidation of pDA to pDQ on the ITO electrode surface, and the second is pDQ reduction by HZ oxidation. We believe that the high anodic current shown in Figure 3 (red line) is governed by the diffusion process of HZ in solution. This is confirmed by the plot of the anodic peak current *versus* square root of the scan rate (Figure 4). As shown in Figure 4B, the anodic peak currents showed good linearity with the square root of scan rate. Therefore, it is clear that the anodic current is certainly generated by the diffusion process of HZ in solution at the pDA-ITO electrode.

### 3.2. Optimization of the Experimental Conditions for HZ Detection

**Figure 5:**
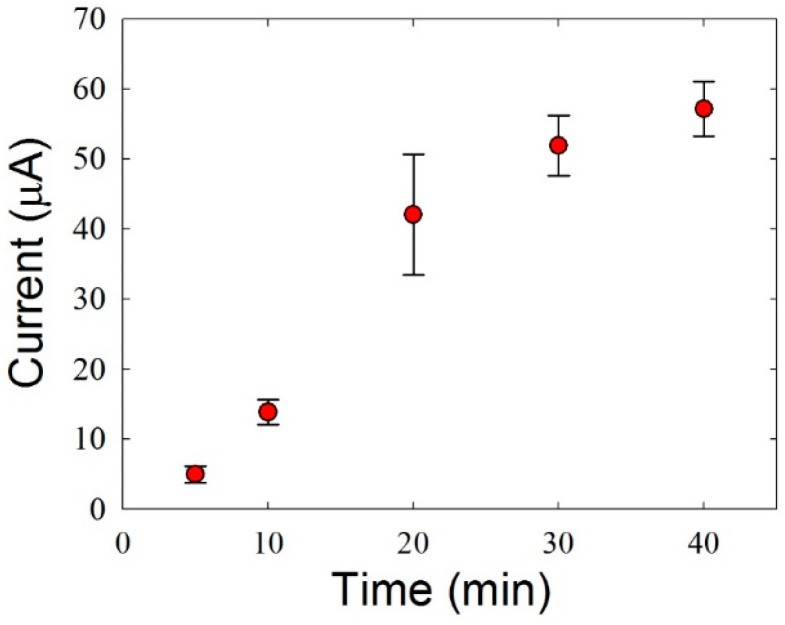
Oxidation currents according to the duration of the submersion of ITO electrodes in 1 mM DA. The error bars indicate the standard deviations of at least three measurements.

To maximize the HZ oxidation current, we varied the amount of DA and the pH of the Tris buffer containing HZ. First, the oxidation currents in 1 mM·HZ were measured with different amounts of DA on the ITO electrodes. The amount of DA can be controlled by varying the duration for which ITO electrodes are submerged in Tris buffer containing 1 mM DA. Figure 5 shows that the HZ oxidation current linearly increases up to a submersion time of 20 min and reaches saturation at 30 min.

**Figure 6:**
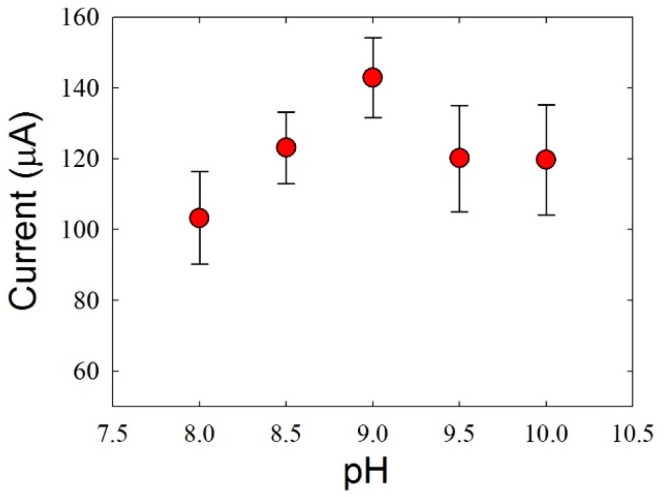
Oxidation currents based on the pH of the Tris buffer. The ITO electrodes were submerged in 1 mM DA for 30 min and then measured in 1 mM·HZ. The error bars indicate the standard deviations of at least three measurements.

Based on this result, we selected 30 min of submersion as the optimal value. Next, the pH of the Tris buffer used for the electrochemical measurement was investigated because the oxidation current of pDA to pDQ can be altered based on the pKa value (8.8) of DA [39]. ITO electrodes submerged in 1 mM DA for 30 min were tested in Tris buffer containing 1 mM·HZ with various pH values. The tested pH values ranged from 8 to 10, and the highest oxidation current was observed at pH 9 (Figure 6). Therefore, Tris buffer with pH 9 was chosen as the optimal test solution for HZ detection.

The subsequent HZ-detection experiments were performed under the optimal conditions of 30 min of ITO electrode submersion in DA solution and measurement in Tris buffer with pH 9.

### 3.3. HZ-Sensing Performances

**Figure 7:**
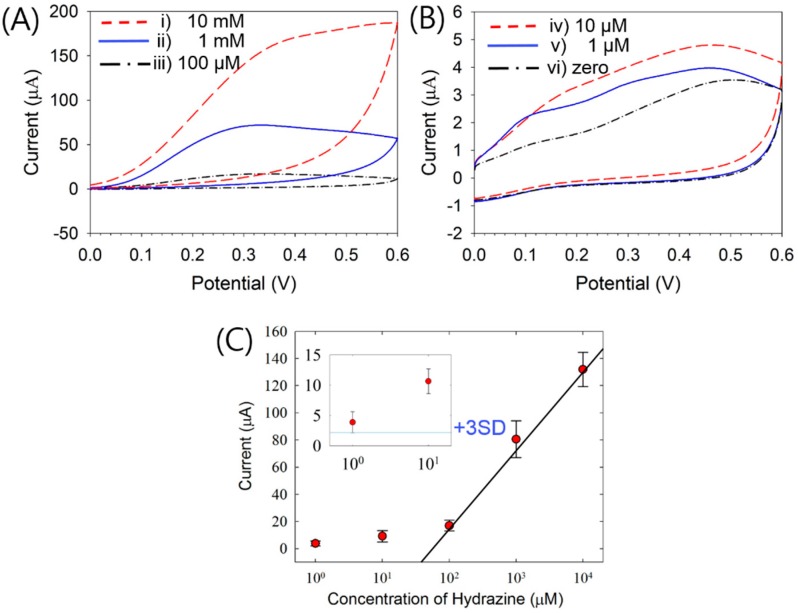
(**A**,**B**) Cyclic voltammograms for various concentrations of HZ in Tris buffer (pH 9) using pDA-ITO electrodes at a scan rate of 50 mV/s: (**A**) (i) 10 mM, (ii) 1 mM, (iii) 100 μM, (**B**) (iv) 10 μM, (v) 1 μM, and vi) zero. (**C**) Calibration plot of anodic currents at 0.3 V. The 3SD line represents the mean current plus three times the standard deviation at 0.3 V for 0 μM HZ. The error bars indicate the standard deviations of six measurements.

Cyclic voltammetry was conducted to determine the concentrations of HZ on the pDA film-modified ITO electrodes at a scan rate of 50 mV/s. The oxidation peak currents at approximately 0.3 V were linearly proportional to the HZ concentration in the range of 100 μM–10 mM (Figure 7A).

The linear relationship between the oxidation currents and HZ concentrations in this range can be described by the following equation (Figure 7C):

*y* = 60.5 log (*x*) (±8.37%) − 101 (±14.9%); (*R*^2^ = 0.9070, 10^2^ ≤ x ≤ 10^4^)

Unlike the case of high concentrations (≥100 μM), another oxidation peak with an ambiguous shape appeared at approximately 0.1 V at low concentrations (≤10 μM) (Figure 7B). We believe that this phenomenon occurred owing to the oxidation of the pDA film when the HZ concentration was lower than the DA concentration [30]. Therefore, we chose to measure the HZ concentration using the oxidation peak current at 0.3 V.

Although the oxidation peak currents increased with the HZ concentration (Figure 7), Figure 7C shows that this relationship was not linear when the concentration was lower than 10 μM. Indeed, the oxidation current of 1 μM HZ shown in the inset of Figure 7C is higher than the 3SD line, indicating that the limit of detection of this method is 1 μM HZ. Therefore, the linear concentration range for HZ detection is 100 μM–10 mM, and the detection limit is 1 μM. These values are comparable to those of other previously reported methods using, for example, nanoparticles [12,40], oxide materials [25,41], and polymers [42] (Table 1).

### 3.4. Influence of Interference Molecules for Detecting HZ and the Stability of pDA-ITO Electrodes

To apply this method to real samples, the pDA-ITO electrodes were used to detect various HZ concentrations in tap water solution with additional ions (containing 50 mM Tris, 300 μM Na^+^, 100 μM Ca^2+^, 100 μM Zn^2+^, 100 μM Mg^2+^, 100 μM Co^2+^, 100 μM Fe^2+^, 600 μM Cl^−^, 100 μM PO_4_^3−^, 100 μM SO_4_^2−^, and 100 μM CO_3_^2−^) (see the Supporting Information, Figure S1). As shown in Table 2, eight samples of tap water, four containing 100 μM and the other four containing 1000 μM HZ, could be detected using the pDA-modified ITO electrode with good recovery (94%–115%).

**Figure 8:**
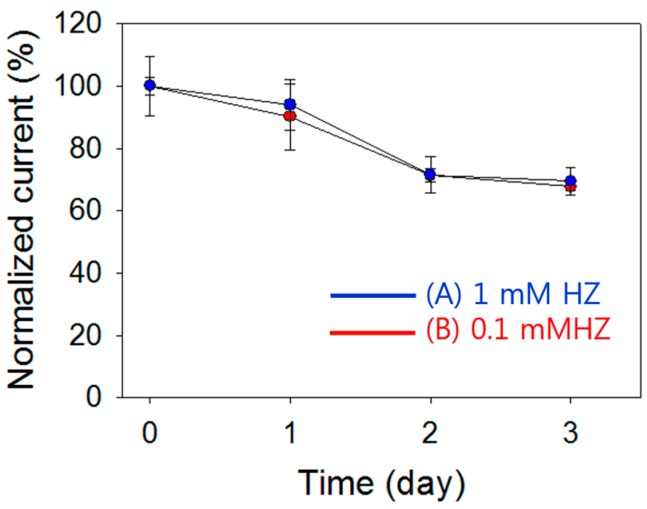
Normalized current (at 0.3 V) for HZ detection according to the time (day). (**A**) 1 mM·HZ and (**B**) 0.1 mM·HZ were detected. The error bars indicate the standard deviations of 4 measurements.

Stabilities of pDA-ITO electrodes were measured with respect to time in a tap water solution containing additional ions (containing 50 mM Tris, 300 μM Na^+^, 100 μM Ca^2+^, 100 μM Zn^2+^, 100 μM Mg^2+^, 100 μM Co^2+^, 100 μM Fe^2+^, 600 μM Cl^−^, 100 μM PO_4_^3−^, 100 μM SO_4_^2−^, and 100 μM CO_3_^2−^). Sixty-four electrodes were prepared on the same day, and four electrodes were used to measure the same amount of HZ each day (tap water solution with additional ions). The prepared electrodes were stored in a refrigerator at 4 °C. As shown in Figure 8, the normalized currents indicated that the detection currents decrease with time. When we used pDA-ITO electrodes 1 day later, 93.9% (±7.96%) and 90.1% (±10.5%) currents were observed when detection experiments were carried out at 1 mM and 0.1 mM·HZ, respectively. After 3 days, the normalized currents decreased to 69.5% (±4.34%) and 67.6% (±1.2%), respectively, for 1 mM and 0.1 mM·HZ. We observed similar decreasing trends in both 1 mM and 0.1 mM·HZ detection experiments. This result means that the pDA-ITO electrodes are not suitable for long-term usage. However, more experiments are needed to extend the stability of pDA-ITO electrodes.

## 4. Conclusions

We have developed a simple and fast EC-based HZ-detection method using pDA film-modified ITO electrodes. The modification of ITO electrodes with pDA films was easily achieved by submerging the electrodes in a DA solution for 30 min. The pDA film acts as a mediator for the EC reaction and shows good performance in the detection of HZ. This HZ detection method has a linear dynamic range of 100 μM–10 mM and has a detection limit of 1 μM. When it is used to analyze tap water containing various concentrations of HZ, it showed good recovery and was not affected by other ionic species present in the samples.

## Figures and Tables

**Table 1:** Comparison of surface-modified electrodes for HZ detection.

Type of Electrode	Detection Limit (μM)	Linear Range (μM)	Sample Condition	Reference
^a^ MnHCF-modified graphite-wax composite	6.65	33.3–8180	Fe(CN)_6_^4−^/Fe(CN)_6_^3^	[9]
CoOOH nanosheets	20	0–1000	0.1 M NaOH	[12]
^b^ CoPc-(CoTPP)_4_/^c^ GCE	230	_	0.2 M NaOH	[22]
^d^ Hb/ZnO/^e^ CNF/GCE	6.60	19.8–1710	0.1 M ^f^ PBS (pH 7)	[25]
SnO_2_/guar-gum hybrid nanocomposite	2760	2000–22,000	Fe(CN)_6_^4−^/Fe(CN)_6_^3^	[40]
Zinc oxide nanorods	~515.7	0.3–300	0.1 M PBS	[41]
Overoxidized polypyrrole	3.7	1.3–2000	0.1 M ammonium buffer (pH 9)	[42]
ZnO-^g^ RGO	0.8	1–33,500	0.1 M NaOH	[43]
ZnO nanoparticles	0.35	0.5–5000	0.01 M PBS (pH 7.5)	[44]
ZnO nanoparticles II	0.147	_	0.01 M PBS (pH 7.0)	[45]
ZnO nanofilm	0.5	0.5–14,200	0.1 M NaOH	[46]
Mn_2_O_3_ nanorods	1.1	2–1300	0.01 M PBS (pH 7.0)	[47]
^h^ PdNPs/^I^ PTAA/GCE	0.00267	0.008–10	0.01 M PBS (pH 7.0)	[48]
^j^ PEDOT:^k^ PSS/Pd	0.12	0.4–100	0.2 M PBS (pH 6.86)	[49]
Pd-^l^GG-g-PAM-silica	4.1	50–180,000	PBS (pH 7.0)	[50]
^m^ AuNPs/^n^ poly(BCP)/° CNT/GCE	0.1	0.5–1000	0.1 M PBS (pH 10.0)	[51]
^p^ PNi-TPPS_4_-NPs	0.11	_	0.1 M NaOH	[52]
^q^ nano-CoTAPC SPEs	30	10–100	PBS (pH 7.4)	[53]
PdNPs/RGO ^r^ RDEs	0.007	0.1–1000	0.2 M PBS (pH 7.4)	[18]
pDA/ITO	1	100–10,000	0.05 M Tris buffer	This work

^a^ MnHCF: Manganese hexacyanoferrate; ^b^ CoPc-(CoTPP)_4_: Cobalt(II)phthalocyanine-cobalt(II)tetraphenylporphyrin pentamer; ^c^ GCE: Glassy carbon electrode; ^d^ Hb: Hemoglobin; ^e^ CNF: Carbon nanofiber; ^f^ PBS: Phosphate-buffered saline; ^g^ RGO: Reduced graphene oxide; ^h^ PdNPs: Pd nanoparticles; ^I^ PTAA: Poly(thiophene-3-acetic acid); ^j^ PEDOT: Poly(3,4-ethylenedioxythiophene); ^k^ PSS: Poly(styrene sulfonate); ^l^ GG-g-PAM: Guar gum grafted with poly(acrylamide); ^m^ AuNPs: Au nanoparticles; ^n^ poly(BCP): Poly(bromocresol purple); ° CNT: carbon nanotube; ^p^ PNi-TPPS_4_: poly-(5,10,15,20-tetra(4-sulfophenyl) porphyrin-nickel); ^q^ nano-CoTAPC SPEs: Cobalt (II) phthalocyanine nanoparticle-modified screen-printed electrodes; ^r^ RDEs: Rotating disk electrodes.

**Table 2:** Determination of HZ in tap water containing ions.

Added (μM)	Sample	Current at 0.3 V (μA)	Found (μM)	Recovery (%) ^a^
100	1	21.5	105	105
	2	20.1	100	100
	3	23.2	112	112
	4	21.1	104	104
1000	1	78.9	940	94
	2	81.3	1030	103
	3	79.6	966	96.6
	4	84.2	1151	115

All solutions comprise tap water containing ions (50 mM Tris, 300 μM Na^+^, 100 μM Ca^2+^, 100 μM Zn^2+^, 100 μM Mg^2+^, 100 μM Co^2+^, 100 μM Fe^2+^, 600 μM Cl^−^, 100 μM PO_4_^3−^, 100 μM SO_4_^2−^, and 100 μM CO_3_^2−^); ^a^ Recovery (%) = (C_Found_/C_Added_) × 100%.

## Supplementary Materials

The following are available online at http://www.mdpi.com/1424-8220/16/5/647/s1, Figure S1. The detail information of cyclic voltammograms in tap water solution with additional ions.

## Abbreviations

The following abbreviations are used in this manuscript:

EPA	Environmental Protection Agency
HZ	Hydrazine
ITO	Indium tin oxide
pDA	Poly(dopamine)
pDQ	Poly(dopamine-*o*-quinone)
