# Supplementary material for: Electrochemical Detection of Hydrazine Using Poly(dopamine)-Modified Electrodes
Source: Sensors (Basel). 2016 May 5;16(5):647. doi: 10.3390/s16050647 (PMC4883338; doi:10.3390/s16050647)
Supplement: Supplementary file 1 [file sensors-16-00647-s001.pdf]

Supplementary Materials: Electrochemical Detection of Hydrazine Using Poly(dopamine)-Modified Electrodes

Ji Young Lee, Truc Ly Nguyen, Jun Hui Park and Byung-Kwon Kim

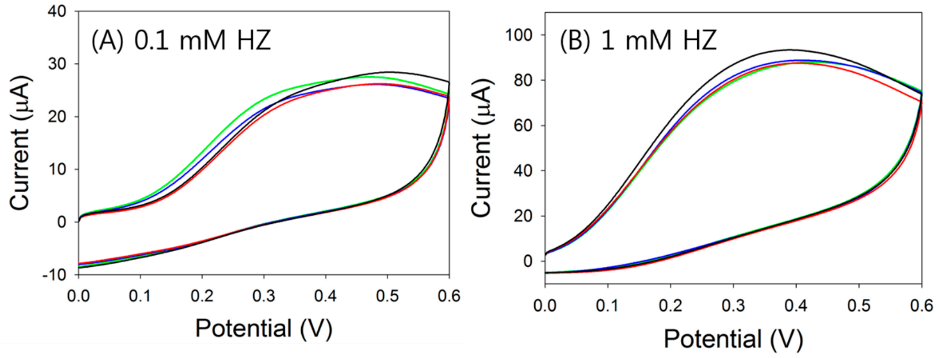

Figure S1. Cyclic voltammograms of (**A**) 0.1 mM·HZ and (**B**) 1 mM·HZ in tap water solution with additional ions (containing 50 mM Tris, 300 μM Na^+, 100 μM Ca^{2+}, 100 μM Zn^{2+}, 100 μM Mg^{2+}, 100 μM Co^{2+}, 100 μM Fe^{2+}, 600 μM Cl^-, 100 μM PO_4^{3-}, 100 μM SO_4^{2-}, 100 μM CO_3^{2-}).
